# Editorial: Storage of Biomass Feedstocks: Risks and Opportunities

**DOI:** 10.3389/fbioe.2021.657342

**Published:** 2021-03-11

**Authors:** Vicki S. Thompson, Timothy A. Volk, Lynn M. Wendt

**Affiliations:** ^1^Biological and Chemical Science and Engineering Department, Idaho National Laboratory, Idaho Falls, ID, United States; ^2^The Sustainable Resources Management Department, State University of New York College of Environmental Science and Forestry, Syracuse, NY, United States

**Keywords:** bioenergy, preservation, degradation, self-heating, composition, ensiling

Storage is a necessary unit operation in the biomass feedstock logistics supply chain, enabling biorefineries to run year-round despite daily, monthly, and seasonal variations in feedstock availability. For example, agricultural sources of biomass such as corn stover are harvested annually and require up to 9 months of storage to enable year-round operation (Darr and Shah, 2012). Industries that rely on forest resources, including the pulp and paper, pellet and bioenergy industries, often store biomass onsite at the processing center for days or weeks to ensure that sufficient material is available (Sahoo et al., 2018). There is much uncertainty about the effect of storage on different feedstocks and for differing utilization approaches. This Research Topic focused on the impact of storage of biomass prior to utilization for bioenergy and/or bio-based products.

At a minimum, effective storage approaches must preserve both the quantity and quality of biomass. Uncontrolled loss of biomass due to microbial degradation is common when storage conditions are not optimized. This can lead to physical and mechanical challenges with biomass handling, size reduction, preprocessing that have negatively impacted demonstration-scale integrated biorefineries (U.S. DOE, 2016). Degradation in storage can also result in biomass that is more recalcitrant to chemical and enzymatic approaches to depolymerization and ultimately results in lower product yields (Groenewold et al., 2020). Loss of feedstock to fires is also possible with dry, combustible feedstocks such as baled material.

The Research Topic was prefaced by a review from Wendt and Zhao that described the state of technology for dry and wet storage systems with a particular focus on improvements that have been observed in feedstocks destined for bioenergy utilization. The article pointed to improvements necessary in the area that can improve stability while maintaining cost competitiveness in comparison to fossil transportation fuels. Nguyen et al. proposed approaches to address this cost barrier by preconditioning biomass during anaerobic storage followed by a fractionation approach to isolate chemically distinct fractions that could have multiple product applications including biofuels, liquid plant biostimulants, and lignin-based phenolic resins for polymers. Such preprocessing is facilitated by biomass depots located near the field to minimize low density transportation costs.

Limiting dry matter loss is one of the most important considerations for storage system design. A study by Therasme et al. examined hot water extraction of wood chips and compared dry matter loss with freshly harvested chips under storage conditions of winter/summer storage, and followed dry matter loss over time and by location in the pile. Dry matter losses were higher during summer storage regardless of treatment. Fresh chips and extracted chips had similar dry matter losses in the initial storage, but extracted chips had much lower losses after 180 days of storage. A model was developed to predict dry matter loss over time in the pile. Quiroz-Arita et al. also developed a model to predict temperature response that results from heat produced during microbial respiration associated with dry matter loss. The model included contributions of conductive and convective heat transfer within a storage zone as well as evaporative heat loss to the environment and thermal capacitance of the biomass itself. Models such as this can be used to understand how temperature increases are indicative of storage losses under aerobic scenarios.

High moisture levels in aerobically stored biomass is directly correlated with dry matter loss due to microbial degradation. A study examining natural air drying with and without added heat was conducted by Mak et al. in western Canada on several types of stored woody biomass. The study demonstrated that positive energy gains could be made relative to the original energy content and that faster drying was possible by only drying during the most favorable conditions.

Smith et al. also investigated the relationship of moisture reduction and dry matter loss in corn stover as a function of aerobic storage in highly insulated storage reactors that mimic bale stacks. The study found that the rate and extent of degradation increased significantly above 36% moisture, wet basis. Stored induced changes were linked to chemical changes due to hemicellulose degradation as well structural changes including increased hydrophilicity, but conversion potential remained unchanged at the biorefinery gate.

Corn stover structural changes occurring in storage were also investigated by Nagle et al. Anaerobic storage through ensiling was utilized to preserve corn stover in long term storage. Minor structural losses in carbohydrates were observed compared to the non-ensiled control; however, bioconversion requirements remained constant. Ultrastructural changes of cell wall matrix removal and re-localization were shown using transmission electron microscopy in ensiled corn stover rind vascular bundles, suggesting that ensiling results in minor changes that may have structural integrity implications in further preprocessing.

Feedstocks applicable to bioenergy systems include agricultural residues (i.e., corn stover, wheat straw), herbaceous energy crops (switchgrass, miscanthus, energy cane, sweet sorghum), woody energy crops (hybrid poplar, coppice willow), forest products and residues, microalgae and macroalgae species, and fractions of municipal solid wastes. Wendt and Zhao suggest storage formats most commonly used for bioenergy resources potentially available in the United States. Wahlen et al. surveyed additional waste resources available in the southern United States that may be compatible with ensiling microalgae. The study then investigated blending grass clippings with microalgae, which preserved dry matter loss while lowering the nitrogen content for downstream thermochemical conversion through hydroprocessing.

A study by Müller and Hahn also investigated blending as a means to preserve biomass in anaerobic storage. Flower strips grown in Europe to enhance biodiversity offer a novel source of biomass available seasonally. The flower strips had modest ability to ensile by themselves but when combined with corn stover, the silage quality was much improved. Additionally, the flower strips contained high levels of nitrate which repressed *Clostridia* activity and preserved dry matter.

In summary, the research represented in the Research Topic exhibited the vast importance of stable storage for bioenergy crops as well as showing how storage-related effects may impact downstream conversion to biofuels or bio-based products through biological/biochemical and thermal/thermochemical and physical deconstruction. Many opportunities exist to use storage to begin to deconstruct the biomass, making it easier to depolymerize prior to conversion.

## Author Contributions

LW and VT wrote the editorial with contributions from TV. All authors approved it for publication.

## Conflict of Interest

The authors declare that the research was conducted in the absence of any commercial or financial relationships that could be construed as a potential conflict of interest.
